# Supplementary material for: Assessing the impact of SARS-CoV-2 prevention measures in Austrian schools using agent-based simulations and cluster tracing data
Source: Nat Commun. 2022 Jan 27;13:554. doi: 10.1038/s41467-022-28170-6 (PMC8795395; doi:10.1038/s41467-022-28170-6)
Supplement: Supplementary file 2 — Reporting Summary [file 41467_2022_28170_MOESM2_ESM.pdf]

nature research

Corresponding author(s): Klimek, Peter

Last updated by author(s): December 3, 2021

Reporting Summary

Nature Research wishes to improve the reproducibility of the work that we publish. This form provides structure for consistency and transparency in reporting. For further information on Nature Research policies, see our Editorial Policies and the Editorial Policy Checklist.

Statistics

For all statistical analyses, confirm that the following items are present in the figure legend, table legend, main text, or Methods section.

n/a	Confirmed	
☐	☒	The exact sample size (*n*) for each experimental group/condition, given as a discrete number and unit of measurement
☒	☐	A statement on whether measurements were taken from distinct samples or whether the same sample was measured repeatedly
☒	☐	The statistical test(s) used AND whether they are one- or two-sided<br>*Only common tests should be described solely by name; describe more complex techniques in the Methods section.*
☒	☐	A description of all covariates tested
☒	☐	A description of any assumptions or corrections, such as tests of normality and adjustment for multiple comparisons
☐	☒	A full description of the statistical parameters including central tendency (e.g. means) or other basic estimates (e.g. regression coefficient) AND variation (e.g. standard deviation) or associated estimates of uncertainty (e.g. confidence intervals)
☒	☐	For null hypothesis testing, the test statistic (e.g. *F*, *t*, *r*) with confidence intervals, effect sizes, degrees of freedom and *P* value noted<br>*Give P values as exact values whenever suitable.*
☒	☐	For Bayesian analysis, information on the choice of priors and Markov chain Monte Carlo settings
☒	☐	For hierarchical and complex designs, identification of the appropriate level for tests and full reporting of outcomes
☒	☐	Estimates of effect sizes (e.g. Cohen's *d*, Pearson's *r*), indicating how they were calculated

Our web collection on statistics for biologists contains articles on many of the points above.

Software and code

Policy information about availability of computer code

Data collection	No software was used to collect data
Data analysis	Custom code used for the analysis is available under https://github.com/JanaLasser/agent_based_COVID_SEIRX/tree/v1.3.0 and https://github.com/JanaLasser/school_SEIRX

For manuscripts utilizing custom algorithms or software that are central to the research but not yet described in published literature, software must be made available to editors and reviewers. We strongly encourage code deposition in a community repository (e.g. GitHub). See the Nature Research guidelines for submitting code & software for further information.

Data

Policy information about availability of data

All manuscripts must include a data availability statement. This statement should provide the following information, where applicable:

- Accession codes, unique identifiers, or web links for publicly available datasets
- A list of figures that have associated raw data
- A description of any restrictions on data availability

The code for the agent based simulation model is openly available under an MIT license at https://github.com/JanaLasser/agent_based_COVID_SEIRX. For the simulations in this publication, version 1.4.1 of the codebase has been used: https://github.com/JanaLasser/agent_based_COVID_SEIRX/releases/tag/v.1.4.1.
The code used to run and analyse simulations is also openly available under an MIT license at https://github.com/JanaLasser/school_SEIRX. For the simulations in this publication, version 1.0.0 has been used: https://github.com/JanaLasser/school_SEIRX/releases/tag/v1.0.0.

The cluster tracing data used to calibrate the model in this study have been deposited at Zenodo under accession code https://doi.org/10.5281/zenodo.4706876.
The data includes all clusters of SARS-CoV-2 infections with at least one transmission in an educational setting recorded between calendar weeks 36 (August 31) and

45 (November 11) 2020 in Austria. Data was collected by Austrian contact tracing agencies in line with the applicable Austrian regulations. No sampling was performed and no data was excluded.
The contact networks and simulation results of this study have been deposited at OSF under accession code https://doi.org/10.17605/OSF.IO/MDE4K.

The following figures have associated raw data (see https://github.com/JanaLasser/school_SEIRX for an in-depth explanation of the data sets and analysis code):
Figure 1 at 10.17605/OSF.IO/MDE4K data/visualization/
Figure 2 at 10.17605/OSF.IO/MDE4K data/contact_networks/calibration/
Figure 3 at 10.17605/OSF.IO/MDE4K data/intervention_measures_delta/
Figure 4 at 10.17605/OSF.IO/MDE4K data/intervention_measures_delta/
Figure 5 at 10.17605/OSF.IO/MDE4K data/sensitivity_analysis/ and data/vaccinations/

Field-specific reporting

Please select the one below that is the best fit for your research. If you are not sure, read the appropriate sections before making your selection.

☐ Life sciences ☒ Behavioural & social sciences ☐ Ecological, evolutionary & environmental sciences

For a reference copy of the document with all sections, see nature.com/documents/nr-reporting-summary-flat.pdf

Behavioural & social sciences study design

All studies must disclose on these points even when the disclosure is negative.

Study description	Quantitative cross-section analysis of cluster tracing data used for calibration in a computational simulation study.
Research sample	Included are all clusters of SARS-CoV-2 infections with at least one transmission in an educational setting, recorded between calendar weeks 36 and 45 in Austria, as identified by cluster analysts of the Austrian Agency for Health and Food Safety. The data does not include completly information on gender of the case. The age of the cases is categorized into age-groups relevant for the school context: <6 years, 6-10 years, 11-14 years, 15-18 years and > 18 years. The distribution of cases in these age brackets is given in Fig. 8 in the main manuscript. The study cases consists cases of the longest time period in which Austrian schools were open with consistent set of measures.
Sampling strategy	No sampling performed.
Data collection	Data collection was performed by the Austrian Food Safety Agency (AGES), which analysed the clusters based on information collected by the PH autorities during contact tracing agencies. Contact tracing is performed using a standardised questionnair by telephone interviews with all laboratory confimred cases of SARS-CoV2 infection. This study re-uses that data which is why a blinded study design is not relevant for the research question we describe in this study.
Timing	August 31, 2020 until November 11, 2020.
Data exclusions	No data was excluded.
Non-participation	Due to the re-use of secondary data, no participants were involved
Randomization	Due to the re-use of secondary data, randomization was not relevant for this study.

Reporting for specific materials, systems and methods

We require information from authors about some types of materials, experimental systems and methods used in many studies. Here, indicate whether each material, system or method listed is relevant to your study. If you are not sure if a list item applies to your research, read the appropriate section before selecting a response.

Materials & experimental systems

n/a	Involved in the study	
☒	☐	Antibodies
☒	☐	Eukaryotic cell lines
☒	☐	Palaeontology and archaeology
☒	☐	Animals and other organisms
☒	☐	Human research participants
☒	☐	Clinical data
☒	☐	Dual use research of concern

Methods

n/a	Involved in the study	
☒	☐	ChIP-seq
☒	☐	Flow cytometry
☒	☐	MRI-based neuroimaging
